# Norwegian Grave Yards

**Published:** 1882-11

**Authors:** 


### NORWEGIAN GRAVEYARDS.

A natural characteristic is the remarkable care that the Norwegians bestow on their graveyards. I stumbled on one at Christiansandt on a Saturday evening, and found it alive with people busily engaged in the work of dressing the plots and ornamenting the graves. The graveyard was not an untidy field, like many of our old churchyards, nor yet was it a stiff and trim garden like most of our modern cemeteries. It was a quiet and secluded rustic retreat in the midst of a little wood, under the shade of ash trees, birches, beeches, and limes. The graves were not mounds covered with green sod; they stood high almost like boxes, and were overgrown with periwinkle and covered on the top with flowers, either growing in the soil or in flower-pots, or set in vases and crystals of various shapes, that of the cross being the most common. The care and neatness with which the graves and grave-plots were kept were quite remarkable. Each family plot formed a little garden, with walks and flower-beds, and generally with a little iron seat at one side of it, where the sorrowing friends might sit and meditate. On nearly every plot one or two ladies were busily engaged in trimming and dressing the garden. They had little rakes in their hands, and also little watering cans, with which they dressed and watered their flowers and shrubs. Fuschias, roses, geraniums, lilies, and other flowers were growing luxuriantly on many graves. This surely is an improvement on the dismal yew trees and the ugly immortelles of basket china to which we are too much accustomed. There can hardly be any fitter or more pleasing emblem of the life beyond than pretty flowers and graceful trees, with their constant life and with their bloom ever being renewed from season to season.—*London Times.*

---

Southern Salt Market.—Mobile has become an important salt market, supplying a portion of the West with that article for various purposes. The salt is brought to that port from Avery's Island, eight or ten miles south of New Iberia, a small town on the celebrated Bayou Teche. The salt is a solid rock mass, assaying ninety-nine per cent. of pure salt. It is without flaw, fissure or seam, and is composed of an aggregation of cubes of one-eighth to three-quarters of an inch. The salt rock weighs 128 pounds to the cubic foot. Wherever struck in the various borings the solid salt always appears directly underlying the surface, and is as solid and pure at its surface as at the bottom of the mine, seventy feet below. The deposit covers an area of 140 acres.

